# Double Mutation in Photosystem II Reaction Centers and Elevated CO_2_ Grant Thermotolerance to Mesophilic Cyanobacterium

**DOI:** 10.1371/journal.pone.0028389

**Published:** 2011-12-22

**Authors:** Jorge Dinamarca, Oksana Shlyk-Kerner, David Kaftan, Eran Goldberg, Alexander Dulebo, Manuel Gidekel, Ana Gutierrez, Avigdor Scherz

**Affiliations:** 1 Department of Plant Sciences, The Weizmann Institute of Science, Rehovot, Israel; 2 Institute of Physical Biology, University of South Bohemia in České Budějovice, Nové Hrady, Czech Republic; 3 Departamento de Producción Agropecuaria, Universidad de La Frontera, Temuco, Chile; 4 VentureLab - Knowledge Center for Business, Universidad Adolfo Ibañez, Santiago, Chile; Louisiana State University and A&M College, United States of America

## Abstract

Photosynthetic biomass production rapidly declines in mesophilic cyanobacteria grown above their physiological temperatures largely due to the imbalance between degradation and repair of the D1 protein subunit of the heat susceptible Photosystem II reaction centers (PSIIRC). Here we show that simultaneous replacement of two conserved residues in the D1 protein of the mesophilic *Synechocystis* sp. PCC 6803, by the analogue residues present in the thermophilic *Thermosynechococcus elongatus*, enables photosynthetic growth, extensive biomass production and markedly enhanced stability and repair rate of PSIIRC for seven days even at 43°C but only at elevated CO_2_ (1%). Under the same conditions, the *Synechocystis* control strain initially presented very slow growth followed by a decline after 3 days. Change in the thylakoid membrane lipids, namely the saturation of the fatty acids is observed upon incubation for the different strains, but only the double mutant shows a concomitant major change of the enthalpy and entropy for the light activated Q_A_^−^→Q_B_ electron transfer, rendering them similar to those of the thermophilic strain. Following these findings, computational chemistry and protein dynamics simulations we propose that the D1 double mutation increases the folding stability of the PSIIRC at elevated temperatures. This, together with the decreased impairment of D1 protein repair under increased CO_2_ concentrations result in the observed photothermal tolerance of the photosynthetic machinery in the double mutant

## Introduction

Photosystem II reaction center (PSIIRC) is a water/quinone oxido-reductase that catalyzes light-activated electron mobilization from the lumenal (water oxidation site) to the stromal (quinone reduction site) side of the photosynthetic membrane. The electrons are transferred through the PSIIRC in a multi-step process initiated by photoexcitation of the primary electron donor (chlorophylls). This process is concluded by the double, stepwise reduction of a mobile quinone, termed Q_B_, by another quinone termed Q_A_. Impairment of the Q_A_^−^→Q_B_ electron transfer steps results in the recombination of electrons and holes, an increased probability of reactive oxygen species production, and attenuation of the PSIIRC repair [1], [2], [3]. The malfunctional PSIIRC undergoes repair that involves its partial disassembly, removal, and proteolysis of the D1 protein subunit, generation of a new D1 protein, and refolding of the repaired PSIIRC to a functional complex [4], [5], [6]. Under physiological light and temperatures, the rates of impairment and repair are balanced, and the steady-state concentration of the PSIIRC maintains continuous photosynthetic activity and growth. Failure to balance the two processes eventually results in cell death [2].

The activity of PSIIRC is highly sensitive to the ambient temperature [7], [8], [9], [10], [11]. Short-term temperature elevation was found to enhance the rate of light-induced oxygen evolution [12]. This phenomenon is reflected by a higher flux of electrons that traverse the PSIIRC complex. The increased flux is probably enabled by the enhanced rate of the Q_B_/Q_B_H2 turnover because of the increased membrane fluidity under short exposure to elevated temperatures and before membrane lipids saturation takes place [13], [14], [15], [16]. The increased flux has a dual effect: [1] it enhances the probability of localized reactive oxygen species (ROS) generation by PSIIRC and the subsequent impairment and degradation of the D1 subunit; [2] the increased supply of electrons to PSI and thereby to the carbon fixation domain results in an increased probability of ROS generation at that site that can lead to the inhibition of protein synthesis and the consequent decrease of the D1 repair activity [17]. The oxidative stress imposed by the increased flow of electrons to the site of CO_2_ fixation is further enhanced by the impairment and reduced activity of Rubisco (ribulose-1,5-bisphosphate carboxylase/oxygenase) at elevated temperatures [18], [19], [20], [21], [22]. Furthermore, at elevated temperatures the affinity of Rubisco for oxygen is increased relative to its affinity for CO_2_ [19], [23], [24] resulting increased photorespiration and overwhelming production of deleterious ROS that impair the D1 repair activity [3], [25], [26].

Short-term (minutes to a few hours) exposure to elevated temperatures results in reversible effects on the photosynthetic activity [27]. However, prolonged exposure (hours to days) to temperatures above the physiological range causes a strong imbalance in the rates of PSIIRC impairment and repair, resulting in the collapse of the photosynthetic machinery and death of the photoautotrophic organism [17], [28]. Therefore, photosynthetic organisms have had to develop strategies to sustain their growth in extremely hot (thermophiles), intermediate (mesophiles), and extremely cold (psychrophiles) habitats. Despite this overall plasticity, the individual strains maintain activity over a narrow range of temperatures, typically ±5–10°C around their physiological optimum. Hence, prolonged global warming is expected to strongly diminish the PSII activity in mesophilic organisms, resulting in reduced biomass production, unstable ecosystems worldwide, as was already observed in oceanic coral populations, and in disruption of renewable energy and food resources [28], [29], [30], [31], [32]. Thus, maintaining a high rate of photosynthesis and biomass formation at elevated, non-physiological temperatures either by increasing the PSIIRC stability or enhancing the rate of D1 repair or both, represent major challenges in acclimatizing photosynthetic mesophiles to global warming [7], [9], [33], [34].

Although many studies have aimed at resolving the role of thylakoids' fatty acid saturation in inducing thermotolerance to photosynthetic organisms [35], [36], we have focused on proteins comprising the PSIIRC. Three major observations provided us with new clues for better understanding the strategy of PSIIRC adaptation to elevated temperatures. First, as we previously showed, the temperature dependence of the first Q_A_^−^→Q_B_ electron transfer rate in mesophiles and thermophiles follows Arrhenius kinetics until it levels off at T*_o_*, which turned-out to be within the physiological temperature range of the examined mesophiles and thermophiles [37]. Second, screening the amino acid sequences in the D1 and D2 subunits of many photosynthetic thermophiles and mesophiles revealed consistent variations in two conserved sites: D1-212 and D1-209, within a GxxxG motif at the protein center [37]. More specifically, D1-Ser212 and D1-Ser209 in mesophiles are replaced by Cys and Ala in thermophiles. Third, single mutations at each of the aforementioned sites could increase the value of T*_o_* by up to 10°C, in line with the observed values in thermophilic strains [37].

In view of the aforementioned observations and considerations we hypothesized that concomitant D1-Ser209Ala and D1-Ser212Cys (AC) mutations may improve the functional stability of PSIIRC in mesophilic cyanobacteria grown at elevated temperatures. We further hypothesized that enhanced CO_2_ concentration should compensate for the increased Rubisco affinity to oxygen at elevated temperatures and thereby reduce the impairment of the D1 repair mechanism by ROS as proposed by Murata *et al* [17] and Takahashi *et al* [26], [28]. All together, we postulated that combination of double mutation and elevated CO_2_ concentration would enable photoautotrophic growth and biomass production at temperatures that cannot be tolerated by the wild type.

The mutations were performed on the ΔKS strain of *Synechocystis* sp. PCC 6803 which was selected as control because it retains only the intact wild-type *psbA*II gene followed by a kanamycin resistance gene (Kmr) [38]. The absence of the other two gene copies, *psbA*I and III genes, which are replaced by spectinomycin (Smr) and chloramphenicol (Cmr) resistance cartridges, simplifies the interpretation of genetic modifications in the D1 protein subunit.

## Results

### The D1-S209A/D1-S212C double mutant (AC) grew photoautotrophically and produced biomass in 1% CO_2_ atmosphere under continuous illumination at 43°C

**Figure 1:**
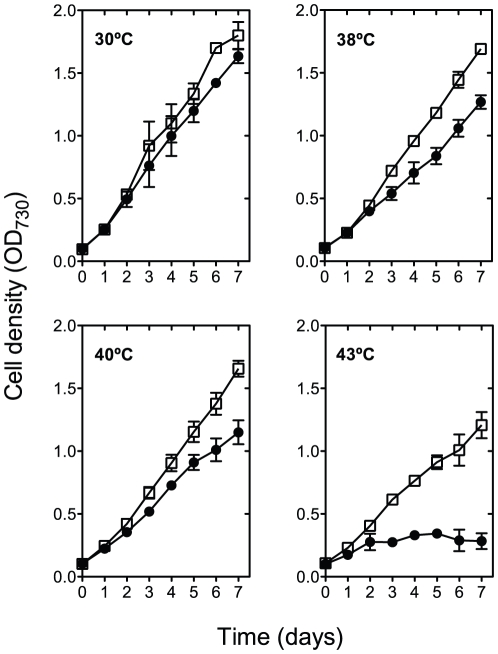
Thermotolerance of *Synechocystis* sp. PCC 6803 AC mutant. ΔKS and AC cells (circles and squares, respectively) were grown in liquid medium for 7 days at 30, 38, 40 and 43°C under 1% CO_2_ aeration and 40 µmol photons m^−2^ s^−1^. Growth was monitored by measuring the optical density at 730 nm (OD_730_). The values represent the mean ± SD of three independent experiments.

**Figure 2:**
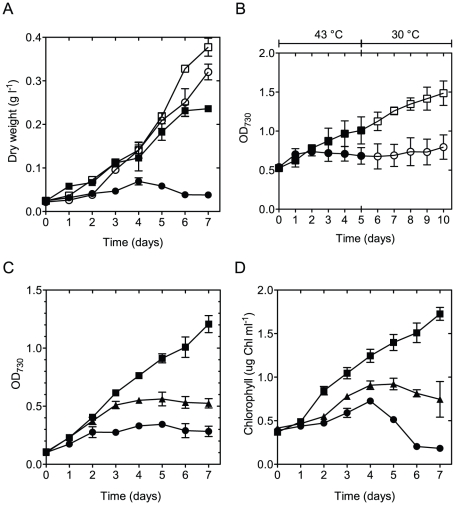
Growth of *Synechocystis* control and AC mutant. Growth was estimated by measuring dry weight (A), OD730 (B and C) and chlorophyll content (D). **A**. ΔKS and AC cells (circles and squares, respectively) that were grown in liquid medium for 7 days at 30° or 43°C (open and filled symbols, respectively) under 1% CO_2_. **B**. ΔKS and AC cultured in liquid medium under stirring and normal air. The strains were incubated at 43°C for 5 days (filled symbols) and then transferred to 30°C (open symbols) for 5 days to test their viability. **C**. Wild-type (triangles), ΔKS (circles) and AC cells (squares) were grown at 43°C under 1% CO_2_. **D**. Chlorophyll content in wild type, ΔKS and AC cells (triangles, circles and squares, respectively) that have been transferred to 43°C and 1% CO_2_ after 3–4 days incubation at 30°C and 1% CO_2_. The values represent the mean ± SD of three independent experiments.

The growth of AC in liquid cultures under continuous illumination (40 µmol photons m^−2^ s^−1^) was monitored and compared with that of ΔKS at 30, 38, 40 and 43°C. There was no significant difference in the growth rates and pigmentation between the two strains when grown at 30°C (Fig. 1) under normal air bubbling or under 1% CO_2_. When incubated at 38 and 40°C the growth of the AC mutant was slightly slower (by 5 and 10% respectively), in comparison to the control strain that showed a much slower growth at both temperatures (by∼25 and 29%, respectively) (Fig. 1). However, when grown at 43°C, ΔKS biomass slightly increased in the first 3 days of incubation followed by complete bleaching after 4 days regardless of the CO_2_ content, whereas the AC mutant, when grown at 43°C and under 1% CO_2_, exhibited a growth rate that was only 25% lower than the one measured at 30°C, resulting in a 15-fold increase in OD_730_ (Fig. 1) and an almost 10-fold increase in the dry biomass after 7 days of incubation (Fig. 2A).

Importantly, when grown at the same temperature but under stirring (no CO_2_ supplement) the AC biomass increased at relatively slow pace and started to level off at the fourth day. Nevertheless, when transferred back to 30°C the growth was regained (Fig. 2B). In contrast, under the same conditions, the ΔKS cultures leveled off after three days of slow growth and could not recover when transferred back to 30°C (Fig. 2B). Thus, although the AC mutant can survive a prolonged incubation at 43°C, normal growth at such temperature requires CO_2_ supplement. Notably, the growth of wild type *Synechocystis* sp. PCC 6803 (having all three psbA genes) at 43°C and 1% CO_2_ showed similar kinetics to that of ΔKS with somewhat higher growth during the first three days of incubation followed by a slower decay from day 4 (Fig. 2C). The chlorophyll (Chl) content in ΔKS and AC increased by 10-fold after 7 days of incubation at 30°C and 1% CO_2_. However, when grown at 43°C, the Chl content sharply declined in ΔKS after 3 days, whereas that in the double mutant increased throughout the entire period of incubation to almost 3 times its initial value (Fig. 2D).

### The D1 and Rubisco proteins exhibited higher steady-state levels in the AC mutant compared with ΔKS, under continuous illumination, 1% CO_2_ and 43°C

**Figure 3:**
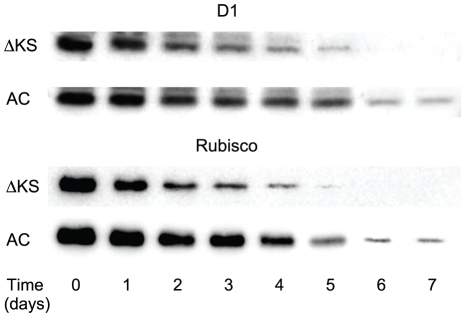
Changes in the D1 and Rubisco large subunit protein content. ΔKS and AC cells were grown in liquid medium at 43°C under 1% CO_2_. Cells were collected at indicated times for isolation of proteins. Samples containing 1 µg of chlorophyll were immunoblotted using antibodies specific against D1 and Rubisco (see Materials and methods).

At 30°C the D1 and Rubisco proteins in both strains maintained a constant steady-state concentration throughout 7 days of incubation under continuous illumination (data not shown). However, upon incubation at 43°C, the D1 and Rubisco proteins content declined ∼2 and 2.2 times faster, respectively, in ΔKS compared with the AC (Fig. 3). Thus, on the fifth day of incubation the D1 content dropped to ∼4% of its initial value (Fig. 3) and that of Rubisco reached non-significant levels for ΔKS. In contrast, at the 6^th^ day of incubation, the AC maintained ∼15 and ∼8% of its initial levels of D1 and Rubisco, respectively. At the 7^th^ day of incubation, no traces of D1 were detected in ΔKS, while ∼8% of its initial level was found in AC (Fig. 3).

### Temperature dependence of the photosynthetic oxygen evolution in cells acclimated at 30 and 43°C under 1% CO_2_

**Figure 4:**
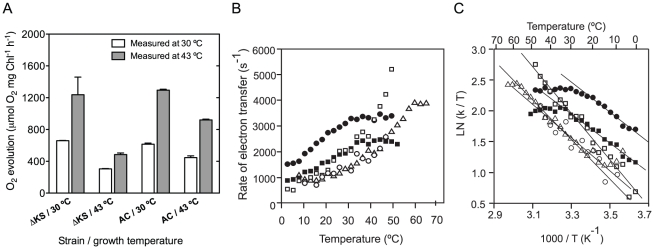
Activity of the PSIIRC in control and AC mutant. Cells were grown for three days at 30° or 43°C (as indicated). **A**. The rate of oxygen evolution was measured at 30°C (white bars) and 43°C (gray bars) after 10 min incubation at the measuring temperature. The values represent the mean ± SD of three independent experiments. **B**. Temperature dependence of the Q_A_^−^→Q_B_ ET rate constant for ΔKS (circles) and AC (squares) grown at 30°C (closed symbols) and 43°C (open symbols). The corresponding curves for *T. elongatus* grown at 43°C are denoted by empty triangles. **C**. The values from B were used to construct the corresponding Eyring plots. The bold lines represent the linear fits of the various curves from which Δ*H* ^‡^ (slope) and Δ*S* ^‡^ (intercept with the Y axis) were derived. For more detailed conditions, see Materials and methods. For **B** *&* **C** the values represent the mean of 10–12 independent measurements, the error bars are not shown here for clarity.

Figure 4A illustrates the temperature dependence of oxygen evolution by the strains grown for three days at 30 or 43°C. Briefly, samples were taken from each culture and then incubated for 10 minutes at the measuring temperature of 30 or 43°C (white and gray bars, respectively). Oxygen evolution by ΔKS and AC grown at 30°C reached similar values when measured at 30°C (659 and 615 µmol O_2_ mgChl^−1^ h^−1^, respectively) with a twofold increase when measured at 43°C (1230 and 1290 µmol O_2_ mgChl^−1^ h^−1^, respectively). A remarkably different behavior was observed for ΔKS and AC grown at 43°C. After 3 days, the oxygen evolution declined in ΔKS to 305 and 485 µmol O_2_ mgChl^−1^ h^−1^ when measured at 30 and 43°C, respectively. The oxygen evolution by the AC mutant was markedly higher after three days of growth at 43°C: 440 and 920 µmol O_2_ mgChl^−1^ h^−1^ when measured at 30 and 43°C, respectively. All together, the rate of oxygen evolution by AC grown at 43°C and measured at 43°C (920 µmol O_2_ mg Chl^−1^ h^−1^) was significantly higher than the activity of the ΔKS grown at 43°C and measured at 43°C (485 µmol O_2_ mg Chl^−1^ h^−1^), suggesting that the PSII activity in the AC mutant underwent acclimation and optimization at 43°C that allowed for retaining activity similar to the one measured under the short term exposure of cells to the elevated temperature, while the control strain did not show such capacity. After 7 days of incubation at 43°C the oxygen evolution activity in the AC mutant approached 20% of its initial value. However, that of ΔKS dropped down to zero already at the fifth day of incubation (data not shown).

### Temperature dependence of the Q_A_^−^→Q_B_ electron transfer rate

The Q_A_^−^→Q_B_ electron transfer rate in ΔKS grown at 30°C leveled off already at 26°C at a value of ∼3400 s^−1^ (Fig. 4B, closed circles) as previously reported [37]. The AC leveled off at a higher temperature of 35°C, though it reached only 2500 s^−1^ (Fig. 4B, closed squares). When grown at 43°C for three days, the small fraction of ΔKS with active PSIIRC (Fig. S1) exhibited a low rate (∼1300 s^−1^) of Q_A_^−^→Q_B_ ET at 30°C that increased to ∼2100 s^−1^ at 43°C (Fig. 4B, open circles). In contrast, after >24 h of acclimation at 43°C, the Q_A_^−^→Q_B_ electron transfer rate in AC increased to ∼3800 s^−1^ when measured at 43°C and continued to rise exponentially reaching a rate of 5200 s^−1^ at 48°C (Fig. 4B, open squares and Fig. S3).

The dissimilarity in the temperature response of the Q_A_^−^→Q_B_ rate constants between the AC mutant grown at 43°C, AC grown at 30°C, and ΔKS grown at 30°C, reflects upon their different enthalpies and entropies of activation for the electron transfer reaction (Fig. 4C and Table 1). More specifically, the activation parameters of the AC mutant acclimated at 43°C (Δ*H* ^‡^ = 7885 cal mol^−1^, Δ*S* ^‡^ = −17.2 cal mol^−1^ K^−1^) become close to those of the thermophilic *T. elongatus* grown at 56°C (Δ*H* ^‡^ = 7142 cal mol^−1^, Δ*S* ^‡^ = −20.7 cal mol^−1^ K^−1^) and are markedly different from those measured for the AC and ΔKS grown at 30°C (Table 1).

**Table 1:** Thermodynamic parameters for the Q_A_^−^→Q_B_ ET.

	ΔKS 30°C	AC 30°C	ΔKS 43°C	AC 43°C	Te 43°C	Te 56°C
Δ*S* ^‡^ (cal mol^−1^ K^−1^)	−25.4	−27.0	−26.2	−17.2	−22.0	−20.7
Δ*H* ^‡^ (cal mol^−1^)	5101.9	4950.2	5404.4	7885.5	6708.0	7142.6

The temperature dependence of Q_A_^−^→Q_B_ ET in ΔKS, AC and *T. elongatus* (Te) cells grown at the indicated temperature was measured as described in Materials and Methods. The activation parameters (Δ*S* ^‡^ Δ*H* ^‡^) were calculated from the Eyring plots (LN(k/T) vs. 1/T) in Figure 4C that represents the mean of 10–12 independent experiments.

### D1 Degradation and Repair

**Figure 5:**
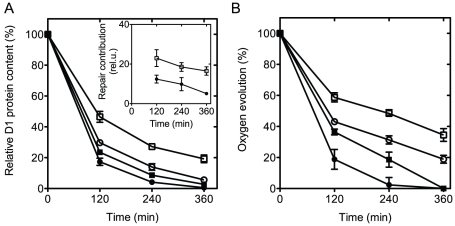
The effect of high irradiance and elevated temperature on D1 protein content and PSII oxygen evolution. The control ΔKS (circles) and AC (squares) cells were incubated at 43°C and illuminated with 500 µmol photons m^−2^ s^−1^ in the absence (open symbols) or presence (closed symbols) of lincomycin. Aliquots of the suspensions were taken at the indicated times. Samples were used for Western blot analysis and to measure the oxygen evolving activity as described in Materials and methods. **A**. D1 protein content. Thylakoid membrane samples were analyzed by SDS-PAGE and immunoblotting using D1-specific antibody. The insert shows the contribution of the repair mechanisms, calculated as the difference between the content of D1 protein in the absence and presence of lincomycin. The data are shown after normalization to the value at the 0 time point. **B**. Oxygen evolution. The oxygen evolving activity was assayed in whole cells. For **A** *&* **B** the values represent the mean ± SD of three independent experiments.

The maintenance of electron transfer and oxygen evolution activity in the AC can be attributed to the higher stability and/or to the enhanced repair rate of their PSIIRCs. To decipher the predominant contribution, we monitored the D1 content as a function of time in cells exposed to high light irradiances, either in the presence or in the absence of the protein synthesis inhibitor lincomycin (Fig. 5A and B).

The D1 protein content in both the AC and ΔKS decayed during incubation at 43°C and under high light conditions. However, the decay of the D1 protein content in the mutant was markedly slower, reaching 20% of its initial value after 6 h of illumination (Fig. 5A). Lincomycin markedly accelerated the decay of D1 content in both ΔKS and AC cultures. Nevertheless, after 6 hours the D1 protein content in the AC reached ∼5–10% of the initial value whereas in ΔKS it dropped to zero (Fig. 5A). Moreover, the difference between the levels of D1 in the absence and presence of lincomycin are ∼2 times larger for the AC compared with ΔKS (Fig. 5A, insert), suggesting that enhanced PSIIRC repair in the AC plays a key role in maintaining high photosynthetic activity at elevated temperatures under elevated CO_2_ concentration.

Under the same conditions, the PSII activity was assessed by measuring the light-saturated steady-state rate of oxygen evolution in the presence of artificial electron acceptors (Fig. 5B). The oxygen evolving activity for both strains decreased during the treatment but the decrease was significantly more pronounced in ΔKS. Thus, after 6 h of exposure, the ΔKS and AC maintained ∼20 and 34% of their initial activity, respectively. However, upon adding lincomycin and thereby preventing PSIIRC repair, ΔKS showed no oxygen evolution already after 4 h of incubation under high light while the AC mutant maintained 35% of its initial oxygen evolution activity at that time. After 6 h of illumination in the presence of lincomycin, the oxygen evolving activity of the AC mutant also dropped to a non-detectable value.

### Conformational changes and related energies that involve inter helical H-bonding of the mutated residues in PSIIRC

*In silico* introduction of the double mutation D1-A209S/D1-C212S to the resolved structure of *T. elongatus* provides insight into the structural and energetic differences between PSIIRC of wild-type *Synechocystis* sp. PCC 6803 and the AC mutant. Hereafter, we will refer to the structure of PSIIRC from *T. elongatus* [39], [40], [41] as representing the putative structure of the AC, and the one obtained by *in silico* D1-A209S/D1-C212S double mutation, as representing the structure of ΔKS *Synechocystis* sp. PCC 6803.

**Figure 6:**
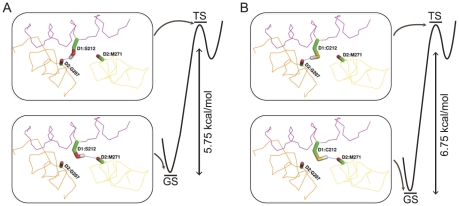
Binding interactions between D1 and D2 proteins. The proposed conformations for the ground (GS, conf 1) and transition (TS, conf2) states of Q_A_^−^→Q_B_ in the *in silico* mutated D1-AC209/212SS (A) and the resolved wild-type structure of *T. elongatus* (B) representing the AC mutant structure.

According to the energy minimized structure, D1-Cys212Sγ enters into an H-bond with the backbone carbonyl of D2-Met271. The DFT computations show that this conformation (Fig. 6B, GS = conf1′) is at an energy minimum. The *in silico* mutation of D1-Cys212 to D1-Ser212 enables a similar ground state conformation, although the D1-Ser212Oγ…D2-Met271 H-bond is longer than the D1-Cys212Oγ…D2-Met271 H-bond (2.63 and 2.17 Å, respectively). The energy for this conformation is ∼1 kcal smaller than the energy for conf1. The second low energy for this *in silico* mutant is one in which D1-Ser212Oγ is H-bonded to the backbone carbonyl of D2-Gly207 at 2.2 Å (Fig. 6A, conf2). The DFT computations show that although the energy of conf2 is at a local minimum, it is ∼5.75 kcal mol^−1^ higher than the energy acquired by conf1. The published structure of *T. elongatus* does not allow for conf2 because the distance between D1-Cys212Sγ and the backbone carbonyl of D2-Gly207 is too short (1.7 Å). Binding at a slightly different geometry (Fig. 6B, conf2′) is possible at an energy that is 6.75 kcal mol^−1^ higher than the conf1 energy for D1-212Cys.

### Molecular dynamics simulations reveal substantial changes in the relative geometries and energies of D helices in PSIIRC during conf1(conf1′)→conf2(conf2′) transitions

**Figure 7:**
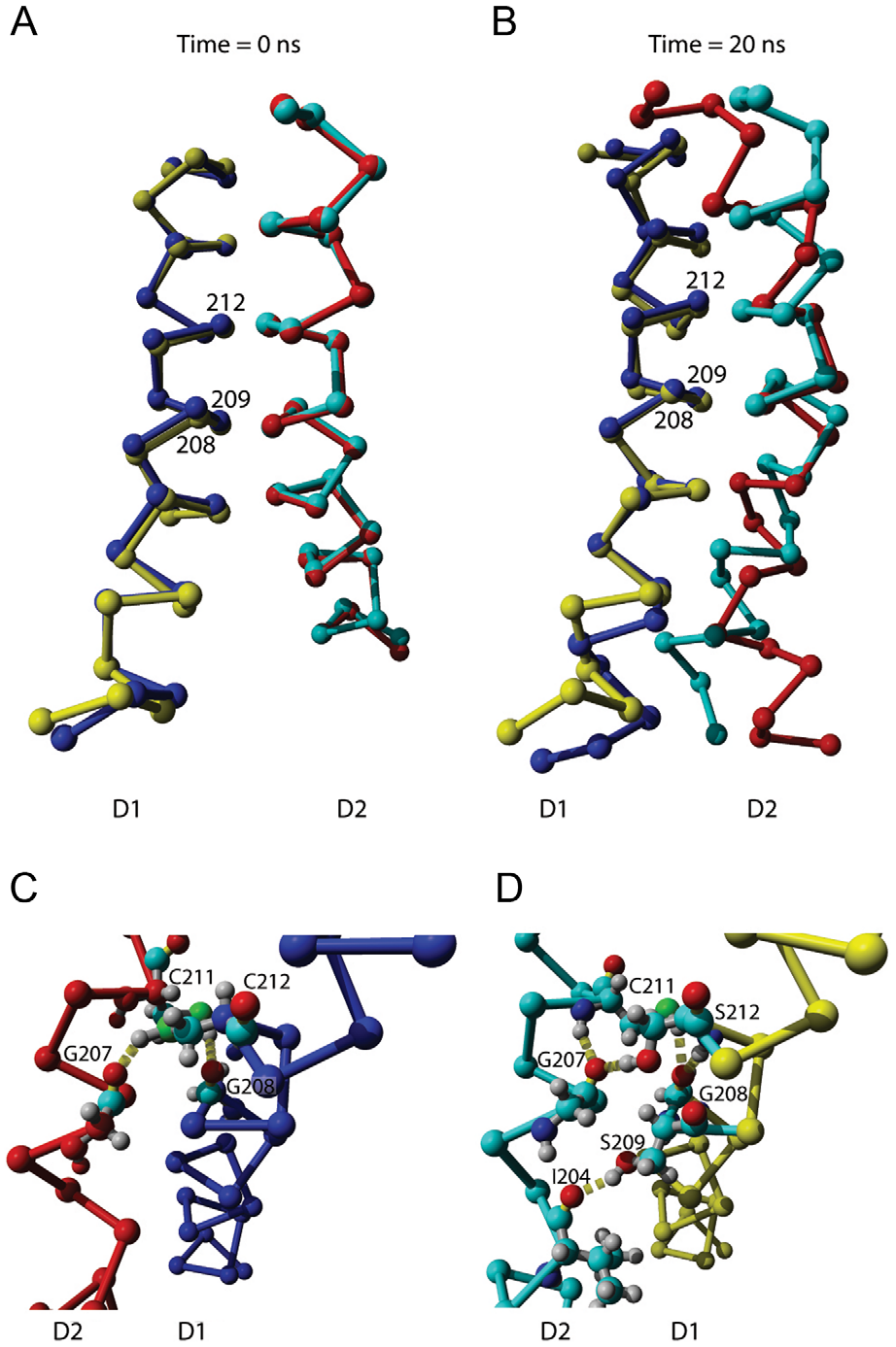
Molecular dynamics simulation of D helix conformations and interactions of the D1 and D2 proteins. **A–D**. Following energy minimization, the C_a_ atoms of the D helices of *Synechocystis* sp. PCC6803 and *T. elongatus* exhibit almost identical structures at the start of the simulation (**A**). Significant deviation is seen mostly on the periphery of the helices at the end of the simulated 20 ns dynamics (**B**). Close up of the central part of the helices shows additional details of the hydrogen bond network in *T. elongatus* (**C**) and *Synechocystis* sp. PCC6803 (**D**). D1 and D2 helices are respectively shown in blue and red for *Synechocystis* sp. PCC6803 and in yellow and cyan for *T. elongatus*.

The molecular dynamics simulations have provided insight into the interaction potential of the D helices of the D1 and D2 proteins (Fig. 7). Two interhelical hydrogen bonds were formed between the D helices of the D1 and D2 proteins of *T. elongatus*; the reciprocal bonding involved the D1-Cys212Sγ donating hydrogen to D2- Gly207O along with D1-Gly208O accepting hydrogen from D2-Cys211Sγ (hydrogen bonding energies were 3.6±1.2 and 3.5±1.3 kcal mol^−1^, respectively, and hydrogen bond lengths were 2.1±0.2 and 2.2±0.2 Å, respectively). In *Synechocystis* sp. PCC6803, the two analogous interhelical bonds D1-Ser212Oγ to D2-Gly207O and D1-Gly208O from D2-Cys211Sγ had hydrogen-bonding energies 5.4±0.9 and 3.9±1.3 kcal mol^−1^, respectively, and the hydrogen bond lengths were 1.8±0.2 and 2.1±0.2 Å, respectively). An additional hydrogen bond was observed between D1-Ser209Oγ and D2-Ile204O (bond length 2.0±0.2 Å, bond energy 4.2±1.4 kcal mol^−1^). As a result of this change in the inter-helical hydrogen bond network, the D helices of the modeled mesophilic D1 and D2 proteins take on a conformation that is different from the thermophilic one. Namely, the average distance of the Cα atoms at the helix-helix interface for ΔKS (d[D1-S212 – D2-G207]) is 6.3±0.3 Å, whereas that for *T. elongatus* or AC (d[D1-C212 – D2-G207]) is 6.6±0.4 Å (Fig. 7). Thus, to form the new H-bond there is a need for a more relaxed environment. Also, the helix-helix contact area in *Synechocystis* hardly increased during the 20 ns simulation, whereas that of *T. elongatus* increased by >2-fold (A_0 ns_ = 165.8 vs. 182.9 Å^2^, A_20 ns_ = 214.4 vs. 428.3 Å^2^ for *Synechocystis* and *T. elongatus*, respectively).

### The effect of temperature on lipids and fatty acid composition in the thylakoid membrane of ΔKS and AC

It is well known that the photosynthetic membrane lipids present increased saturation with increasing temperature [42], [43], [44] in different strains of cyanobacteria and higher organisms. Such a change may affect the energy required for the D1 and D2 subunits to undergo the conformational changes required to enable the gating of the Q_A_^−^→Q_B_ electron transfer which was observed for the AC strain following >24 h of incubation at 43°C. Hence, we followed the timeline for the increased saturation of the thylakoid membranes in ΔKS and AC and compared it with the observed changes in the thermodynamic parameters for the electron transfer (Table S1 and Table 2). In both ΔKS and AC the double bond index (DBI) value decreased with increasing temperature and reached constant value after 24 h of incubation. This included a similar increase of the MGDG/DGDG ratio (Table S1). Importantly, the fatty acid composition in ΔKS that grew at 30°C was found to be similar to the one previously reported for *Synechocystis* sp. PCC 6803 [36], [45], with a relatively high percentage of polyenic acids that provided a fairly high DBI value (96.8). The DBI value in the AC mutant that was grown at 30°C was higher (104.6), mainly because of an increase of linoleic (18∶2) and a decrease of palmitic (16∶0) fatty acid contents, reflecting a more fluid membrane environment at standard growing conditions.

**Table 2:** Fatty acids in thylakoid membranes from ΔKS and AC mutant cells grown at different temperatures.

Strain	Temperature	Fatty acids										
		16∶0	16∶1	16∶2	18∶0	18∶1c	18∶1t	18∶2	18∶3 n3	18∶3 n6	18∶4	DBI
**ΔKS**	30°C	53.4	10.2	0.3	0.4	4.8	0.4	10.9	b–	19.1	0.4	96.8
	43°C	58.3	6.6	0.3	0.6	9.2	0.8	11.4	1.2	11.4	0.3	78.9
**AC**	30°C	49.6	10.3	0.6	0.3	5.9	0.4	12.3	–	20.0	0.6	104.8
	43°C	53.3	6.7	0.4	0.3	13.1	atr.	12.9	1.0	11.9	0.3	86.2

Cells were grown at the indicated temperature for three days. Total lipids were extracted from thylakoid membranes and fatty acids were analyzed as described in Materials and Methods. The double-bond index (DBI) is the sum of percentages of unsaturated fatty acids multiplied by total number of double bonds. The values are the means of three independent experiments and are expressed as mol %. The deviation of values was within ± 2%.

a tr. Trace amount (less than 0.2%).

b -. Non-detected.

Incubation of ΔKS and AC mutant at 43°C for >24 h was sufficient to decrease their DBI values to 78.9 and 86.2, respectively. A similar but somewhat lower value was reported by others for *Synechocystis* sp. PCC 6803 grown at 38°C [36]. The lower DBI is mainly the result of elevated levels of saturated palmitic (16∶0) and a decrease of both unsaturated palmitoleic (16∶1) and polyunsaturated gamma-linolenic (18∶3 n6) fatty acid contents. Even though both strains showed a lower DBI after growth at 43°C, the AC exhibited a higher value than ΔKS, which reflects a higher fluidity in the thylakoid membrane.

## Discussion

The goal of our research is to genetically engineer novel, mesophilic cyanobacteria that retain prolonged photosynthetic activity and biomass production under continuous illumination at elevated temperatures to which the “wild type” cannot adjust. We hypothesized that PSIIRC should be a major target for such engineering and that genetic differences between the PSIIRC in thermophiles and mesophiles provide clues for new strategies. Following sequence alignment analysis, we found two sites within a GxxxG-like motif in the D1 protein subunit that are consistently occupied by different residues in thermophiles and mesophiles. In a previous study, we focused on the effect of single mutation on the electron transfer dynamic with respect to the ambient temperature [37]. Here, we aimed at deciphering the mutations effect on the bacterium viability at above its physiological temperature. Therefore, a double mutation was performed in the GxxxG-like motif, which made it identical to the one found in the thermophilic cyanobacterium *T. elongatus*. Indeed, only a slight decrease in the growth rate was shown by the AC mutant at 38 and 40°C, temperatures at which the ΔKS grew at a much slower rate. More important, the double mutant presented prolonged photosynthetic activity and biomass growth during 7 days incubation at 43°C far above the growth temperature of wild-type *Synechocystis* sp. PCC 6803 or the ΔKS strain that was used as control, but only under elevated CO_2_ conditions (1%). The ΔKS completely perished already after 6 days of incubation under identical growth conditions.

Notably, when ΔKS and AC mutant cells were grown at 43°C but at lower CO_2_ supply both strains showed slower growth. However, in all tested CO_2_ supply conditions (stirring, air bubbling and 1% CO_2_) the AC mutant showed higher growth than the ΔKS (Fig. S2). Moreover, when the CO_2_ concentration was increased to 3% the AC mutant showed growth even at 45°C, while the ΔKS grew only for 1 day (Fig. S2). The beneficial effect of the increased CO_2_ concentrations could be attributed to two mechanisms. First, the increased affinity of Rubisco to oxygen at elevated temperatures decreases CO_2_ fixation, which can subsequently increase the generation of ROS [19], [23], [24]. Furthermore, the reduction of molecular oxygen may lead into the formation of H_2_O_2_ that inhibits the synthesis of PSII proteins and primarily of the D1 protein [3], [25], [26]. Second, CO_2_ solubility drops by estimated 30% upon increasing temperature from 30 to 45°C, further increasing the probability of Rubisco to react with oxygen. Thus, the increased CO_2_ concentration should help decreasing the probability of oxygen binding to the Rubisco and consequently the formation of radicals that could impair the D1 repair activity.

The longevity of the double mutant at the high temperature and elevated CO_2_ conditions, appears to be correlated with the markedly slower decline of the PSIIRC activity monitored by the levels of electron transfer and oxygen evolution activities as well as the level of the D1 protein subunit and Rubisco (Fig. 3 and 4). The relatively larger amplitude of the fastest component in the fluorescence decay curves (Fig. S1) provides an additional support for the enhanced functional stability of the PSIIRC in the AC mutant. Importantly, only when the level of D1 and Rubisco reach <20% and 5–10%, respectively, of the content found at room temperature, the rate of biomass growth slowed down (Fig. 1 and 3). This finding is in agreement with previous studies reporting that photosynthetic organisms with less than 50% of their steady state D1 level can still maintain the same rate of biomass formation because of the excess capacity of light-induced electron transfer in PSII [46], [47]. Nevertheless, maintaining a normal growth rate, even at 10–20% Rubisco protein level, is an interesting finding that requires further investigation.

To elucidate the contributions of enhanced PSIIRC photo thermal stability and the rate of repair to the subunit steady-state concentration, we followed the decay of the D1 protein content and the concomitant PSII activity during exposure to high irradiance at 43°C in the presence and absence of lincomycin. The difference between the respective pair of measurements (represented by full and empty symbols, respectively, in Fig. 5) represents the contribution of D1 and PSIIRC repair to the measured quantity, as demonstrated by the insert in Fig. 5A. In both strains, the D1 protein content and oxygen evolution activity exhibited a decrease during the exposure and this decrease was enhanced in the presence of lincomycin (Fig. 5A and B). Nevertheless, the AC mutant showed higher content of D1 protein and higher activity than the ΔKS during the course of the treatment both in the absence and presence of lincomycin. These results suggest that both the stability (Fig. 5A and B) and repair (Fig. 5A, insert) of the D1 protein are enhanced in the double mutant compared with ΔKS. The increased rate of repair in the AC mutant may reflect upon increasing rate of PSIIRC refolding with the mutated D1 protein or, upon higher photo/thermal stability of the mutated psbA mRNA. This question is currently being explored in our lab.

We previously showed that the rate of Q_A_^−^→Q_B_ electron transfer levels off at *T_o_*, which was defined as the optimal temperature for electron transfer, and it was found to be within the physiological range of the examined strain, 26°C for ΔKS and ∼59°C for *T. elongatus* [37]. Importantly, the rate of Q_A_^−^→Q_B_ electron transfer at *T_o_* is similar for mesophiles and thermophiles, reaching a value of 3000–4000 s^−1^. Apparently, at this rate the balance between PSIIRC degradation and repair, as well as other enzymatic processes that comprise photosynthetic charge separation and carbon fixation, is optimal. This observation is in line with the corresponding hypothesis, which suggests that psychrophilic (cold-adapted), mesophilic, thermophilic and hyperthermophilic homologous enzymes have comparable catalytic efficiencies (indicated by kcat/K_M_) at their respective optimal temperatures because optimal activity requires a certain degree of conformational flexibility in the active site [48], [49]. Hence, one can reasonably assume that for inducing thermotolerance and biomass generation, as has been sought in this study, Q_A_^−^→Q_B_ electron transfer needs to reach and maintain a value of ∼3500 s^−1^ when growing at the elevated target temperature. Following the transition state theory, the rate of Q_A_^−^→Q_B_ electron transfer is given by (*k_et_*) = (k_b_T/h)exp(−Δ*E^‡^*/*RT*), where k_b_ and h represent the Boltzmann and Max-Plank constants, respectively, and Δ*E^‡^* = Δ*H^‡^−T*Δ*S^‡^* is the activation energy [37]. Thus, maintenance of similar electron transfer rates at the physiological optima of mesophiles and thermotolerant/thermophiles can be achieved by adjusting the Δ*E^‡^*, or more specifically, the Δ*H^‡^* and Δ*S^‡^* values, to the ambient temperatures. As shown above, the Δ*H^‡^* and Δ*S^‡^* values for AC during prolonged incubation at 30°C are slightly different from those of ΔKS and therefore *k_et_* reaches maximal value and levels at ∼33°C (Fig. 4*C* and Table 1). However, after ≥24 h of acclimation at 43°C, *ΔH^‡^* and *ΔS^‡^* of the AC are changed by +3 kcal mol^−1^ and −9.8 cal mol^−1^ K^−1^, respectively (Table 1). With these new values, *k_et_* is equal to 3500 s^−1^ only when reaching T = 42°C, which becomes the new optimal temperature (*T_o_*) for PSIIRC activity. Likewise, provided that the optimal rate for electron transfer in *T. elongatus* is also ∼3500 s^−1^, (Fig. 4*C*) acclimation at 43°C results in *ΔH^‡^* and *ΔS^‡^* values that provide the optimal *k_et_* = 3500 s^−1^ at *T_o_* = 52°C, whereas growth at 56°C acclimatizes the strain for *k_et_* = 3500 s^−1^ at *T_o_* = 57°C. Cumulatively, the change in thermodynamic data after >24 h of incubation combined with the DFT calculations and dynamic simulations suggest that the PSIIRC ground state is more stable by at least 2 kcal mol^−1^ in the AC (Fig. 6).

The change in the Q_A_^−^→Q_B_ electron transfer rate and the related thermodynamic parameters, appears to occur after >24 h of incubation at 43°C under 1% CO_2_ (Fig. S3), this appears to correlate with the timeline for increased saturation of the membrane lipids in *Synechocystis*, already reported by others [36], [44]. Loll et al (2007) identified six MGDG, four DGDG, three SQDG, one PG as well as three β-DM molecules per PSIIRC monomer. The most recent structure [50] reports 1 more DGDG (5 in total), 1 more SQDG (4 in total) and 4 more PG (5 in total). According to Sakurai *et al* (2006), only MGDG and DGDG contain the 18∶3 fatty acids that undergo saturation and consequently rigidification, similarly to the trend observed here upon incubating ΔKS and AC at 43°C (Table 2). Moreover, the percentage of these lipids in thylakoid membranes and isolated PSIIRC is practically the same [45], [51]. Hence, the MGDG and DGDG, which interact with PSIIRC, may experience the aforementioned 18∶3→18∶1 and 16∶1→16∶0 transition under prolonged temperature elevation. The resulting rigidification of the lipids interacting with D1/D2 proteins should modify any process involving a protein conformational change that requires displacement of these lipids. The possibility of such conformational changes in the studied strains is discussed in the following text.

The activation energy for the Q_A_^−^→Q_B_ electron transfer process is indicative of dissociation of 1–2 H-bonds [37] upon transferring from a ground to a transition state during the Q_A_^−^→Q_B_ electron transfer. The molecular dynamic simulations (Fig. 7) indicate that alternating H-bonding association or dissociation of the D1 and D2 protein subunits involve an average 0.7–1 Å expansion of the D1/D2 complex in the AC but not in ΔKS. At a high level of desaturation (e.g. following incubation at 30°C), reflected by a high DBI value (Table 2), the flexible lipid bed provides similar low resistance to conformational changes of the D1/D2 in ΔKS and the AC. The higher DBI value for AC compared to ΔKS, possibly reflect upon the larger membrane flexibility that is needed to allow the larger PSIIRC expansion during electron transfer. However, following acclimation at elevated temperatures, the increased saturation makes the lipid environment of the PSIIRC more rigid than at room temperature and the conformational change in the AC needs to overcome an additional energy barrier accounting for part of the markedly increased Δ*H* ^‡^. The enhanced rigidity of the lipids should decrease the entropy of the Q_A_^−^Q_B_ state and therefore the value of Δ*S* ^‡^ for the Q_A_^−^→Q_B_ transition is reduced. The experimental values of the thermodynamic parameters (Table 1) fit the putative conformational changes and the involved energies that are presented in Figure 6, following the DFT computations. Thus the thermodynamic parameters show both stabilization of the ground state and enhanced conformational rigidity of the AC after acclimation at the elevated temperature. Such enhancement is often proposed to account for the increased thermal stability of thermophilic enzymes compared with mesophiles [52].

The significance of fatty acid saturation in regulating enzymatic reactions that depend on protein conformational changes, was explored using lipid specificity for the reconstitution of well-coupled ATPase proteoliposomes [53]. Different approaches attempted to decipher the role of saturation/desaturation in adapting the photosynthetic machinery to temperature changes (for a recent review see: Allakhverdiev et al, 2008). However, a recent study claims that membrane protein stability does not depend on the lipid composition of the membrane [54] and arguments against lipid saturation per se as a regulator of thermotolerance were raised [36]. The present study supports the possibility that both lipid saturation/rigidification and point mutations that modify the protein structure at the transition state may be required for controlling the activation energy for the rate-determining electron transfer and for inducing thermotolerance to the PSIIRC. Namely, as the growth temperature is increased to 43°C, Δ*H^‡^* and Δ*S^‡^* for the AC mutant should be changed for maintaining *k_et_* at 3000–3500 s^−1^. To that end the DBI value decreases to counteract the increased fluidity of the membrane at elevated temperatures.

At the same time our study suggests that in addition to the enhanced functional stabilization of the PSIIRC complex, there is a need to attenuate the impairment of the D1 repair machinery at elevated temperature, possibly by providing more CO_2_ as a sink for the accumulation of redox equivalents.

Furthermore, the sequence, structure, and thermodynamic similarities between the PSIIRC in the AC and *T. elongatus* suggest that the AC double mutation could account for the thermophilicity of existing strains and could provide a first step for adapting mesophilic photosynthetic organisms.

## Materials and Methods

### Growth conditions and treatments

Stock cultures of the control strain ΔKS and the double mutant D1-S209A/D1-S212C (hereafter, AC) were grown photoautotrophically at 30°C in BG-11 medium under continuous illumination with aeration of 1.0% CO_2_ in air (1 l min^−1^). Cultures of similar cell density were incubated at 30, 38, 40 or 43°C under 40 µmol photons m^−2^ s^−1^ white light. Growth was monitored by measuring the optical density of the culture at 730 nm (OD_730_) and the dry weight biomass. Changes in chlorophyll concentration were measured spectroscopically by sampling aliquots from the liquid cultures every 24 hours as previously described [55]. In some experiments, the protein synthesis inhibitor lincomycin was added to the cell suspension (final concentration 200 µg ml^−1^) at the start of the treatment [6]. *Thermosynechococcus elongatus* BP-1 [56] cells were grown at 30, 43 or 56°C under a light intensity of 40 µmol photons m^−2^ s^−1^ white light in liquid BG-11 medium.

### PCR-based mutagenesis

Mutagenesis on the ΔKS strain was performed as previously described [37] with the following modifications. The S209/212 primer 5′-GGT GGA TTC GGT GGT **GCC** TTG TTC **TGT** GCC ATG CAT GGT TCC-3′ was prepared to insert mutations at bp 625–627, corresponding to the aminoacid D1-Ser209 and at bp 634–636, corresponding to D1-Ser212 [57], to obtain the D1-S209A/D1-S212C double mutant.

### Isolation of proteins and Western blot analysis

Thylakoid membranes were prepared as previously described [58]. Whole-cell extract samples were obtained from the same preparation and used for Rubisco Western blot analysis. Proteins were solubilized in sample buffer (0.5 M Tris-HCl pH 6.8, 1% SDS, 24% glycerol, 4% β-mercaptoethanol, 0.001% (w/v) Bromophenol blue), incubated at room temperature for 1 hour and then separated on 12.5% SDS-PAGE. The equivalent of 1 µg of chlorophyll was loaded in each well. Proteins were electroblotted to PVDF (Hybond-P, Amersham, UK) using a BioRad Mini Transblot Cell (Bio-Rad, USA). The immuno detection was carried out using a chemiluminescence kit (SuperSignal West Pico, Pierce, USA). Antibodies against D1 and RuBisCO large subunit proteins were purchased from Agrisera (Umeå, Sweden). For quantification the bands from the scanned blots were quantified by integrating variable pixel intensities using the ImageJ software [59] and comparing them to a dilution series of samples (Fig. S4).

### Oxygen evolution rate

Light-saturated (1500 µmol photons m^−2^ s^−1^) steady-state rate of oxygen evolution was measured using a Clark-type platinum silver electrode in a thermostated glass cuvette (Hansatech, Inc., England). Cells were harvested at the indicated times by centrifugation and re-suspended in fresh media to a final concentration of 10 µg chlorophyll ml^−1^. The cells were kept in the dark for 10 min at the measuring temperature before being measured. A total of 3 ml of cells was added to the electrode chamber and the artificial electron acceptors 2,5-dimethyl-p-benzoquinone (DMBQ) (0.5 mM final concentration) and ferricyanide (1 mM final concentration) were added just before measurement. The temperature was maintained at 30 or 43°C with a circulating water bath. Calibrations at both measuring temperatures were made to adjust the sensitivity of the electrode.

### Extraction, preparation, and analysis of thylakoid membrane lipids and their fatty acids

Thylakoid membranes were isolated as described [60]. Thylakoid lipid extraction was carried out according to [61]. Lipid classes were separated by thin-layer chromatography on silica gel (Merck 5721) with chloroform/methanol/acetic acid/water (90∶9∶12∶2 v/v) as the developing solvent. TLC plates were sprayed with 0.05% solution of primuline. Lipid spots were visualized with a hand-held UV lamp VL-6.M (Vilber Loumart, France) in order to assess the quality of separation or to mark lipids for scraping and extraction. The plates were then scanned and the spots were quantified by integrating variable pixel intensities on ImageJ software [59] and comparing them to standard curves. Lipid spots on the TLC plates were scrapped-off and later subjected to transmethylation [62]. The esterified fatty acids were analyzed with a gas-liquid chromatograph (HRGC 5300, Carlo Erba instruments) equipped with a hydrogen flame-ionization detector. The double bond index (DBI) was calculated by dividing the sum of the percentages of the unsaturated fatty acids, each multiplied by the number of its double bonds, by 100.

### Flash Fluorescence Measurements

The rate constant for the first Q_A_ to Q_B_ electron transfer was assessed as recently described [37]. The percentage of active PSIIRC was deduced from the relative contribution of the fast component to the overall decay of the intact cell fluorescence as previously described [27], [37], [63].

### DFT calculation and protein dynamics

PSII structural coordinates at 2.9 Å resolution (PDB ID: 3bz1, [40]) were downloaded from the PDB and hydrogen atoms were added using REDUCE [64]. Relevant parts of helices D and E from D1 (aa 203–217, aa 268–281) and D2 (aa 202–216, aa 264–277) were extracted and capped with hydrogen atoms at the N- and C-termini; only this four-helix bundle was considered in the following calculations since D1-C212 does not interact with other parts of the protein. The possible H-bonds formed by this cysteine's side-chain were determined by optimizing its Sγ, Hγ, 1Hβ, and 2Hβ atoms (all other atoms were frozen) at 12 initial side-chain conformations, defined by combinations of χ1 (180°, +60°, −60°) and χ2 (180°, +90°, 0°, −90°) dihedral angles. These 12 optimizations resulted in a global minimum and several local minima. A similar procedure was applied for D1-C212S by adjusting the bond lengths and angles of its Oγ. For an accurate calculation of the H-bond geometries and energies, while considering the four surrounding helices, a quantum mechanics/molecular mechanics (QM/MM) hybrid method was utilized. Specifically, a two-layer ONIOM approach [65], as implemented in GAUSSIAN 03 [66], was applied. The QM layer for the D1-C212 (and D1-S212) optimizations consisted of the following atoms: Cα, Hα, Cβ, (2X)Hβ, Sγ (or Oγ), and Hγ of D1-C212; Cα, (2X)Hα, C, and O of D2-G207; N and H of D2-A208 and D2-C211; Cα, Hα, C, and O of D2-M271; N and H of D2-L272; N, Cδ, (2X)Hδ, Cγ, and (2X)Hγ of D2-P275. The DFT-B3LYP/6-31+G** level of theory was used for the QM layer. The hybrid B3LYP [67], [68] functional was used as an intermediate-level means of including electron correlation, since it has been shown to produce accurate geometries compared with protein structures [69] and realistic H-bond energies in ligand protein systems [70]. The double-ζ 6-31+G** basis set contains polarization functions on all atoms and diffuse functions on heavy atoms. The MM layer, consisting of all other atoms, was modeled by the Amber force field [71], and its partial charges were able to polarize the QM wavefunction (electronic embedding).

We did not find significant differences in the results when using the most recent PSII crystal structure [50].

#### Molecular dynamics simulations

The simulations were performed with YASARA [72]. The 3D structures of the D helices of the D1 and D2 proteins in thermophilic *Thermosynechococcus elongatus* were obtained from the crystal structure of cyanobacterial PSII (PDB ID: 3BZ1) [40] D1(196–221): PFHQLG VAGVFGGALFCAMHGSLVTS; D2(195–219): PFHMMGVAGVLGGALLCAIHGATVE. The *in silico* mutagenesis of two amino acid residues in the D1 sequence - A209S and C212S (underlined) mimicked the native structure of the mesophilic *Synechocystis* sp. PCC6803. The D2 sequence of the D helix differs only at the site 204 – the thermophile valin was replaced for isoleucine in the mesophile. Both models of the mesophilic and thermophilic D helices of the D1 and D2 proteins were placed in the periodic boundary simulation boxes that were 1 nm larger than the peptides along all three axes. After hydrogen atoms were added to the helices according to basic chemistry rules and the currently selected pH = 8.0, the boxes were filled with TIP3P water, and sodium atoms were iteratively placed at the coordinates with the lowest electrostatic potential until the cell was neutral. Molecular dynamics simulations were run using a multiple time step of 1.25 fs for intra-molecular and 2.5 fs for intermolecular forces. To remove bumps and to correct the covalent geometry, the structures were energy-minimized with the Yamber3 force field [73] using a 8.0 Å force cutoff and the Particle Mesh Ewald algorithm [74] to treat long-range electrostatic interactions. After removal of conformational stress by a short steepest descent minimization, the procedure was continued by simulated annealing (time step 2 fs, atom velocities scaled down by 0.9 every 10th step) until convergence was reached, i.e., the energy improved by less than 0.012 kcal mol^−1^ during 200 steps. The simulations were then run at 300 K at a constant pressure (NPT ensemble) to account for volume changes due to fluctuations of peptides in the solution. The simulations were run for a total time of 20 ns. Molecular graphics were created with YASARA [72] and Persistence of Vision (TM) Raytracer (http://www.povray.org/).

## Supporting Information

Figure S1 **Amplitudes of the fast decay component of chlorophyll fluorescence.** The amplitudes represent the relative contribution of PSIIRCs that perform normal Q_A_^−^→Q_B_ electron transfer to the total PSIIRC content. Fluorescence was measured in ΔKS grown at 30°C (open circles), ΔKS grown at 43°C (filled circles), AC grown at 30°C (open squares), AC grown at 43°C (filled squares) and *T. elongatus* grown at 43°C (open triangles). The values represent the mean of at least 10 independent experiments; the error bars are not shown here for clarity.
(EPS)

Figure S2 **Photoautotrophic growth of the ΔKS and AC mutant cells at elevated temperatures and various CO_2_ concentrations.** ΔKS (circles) and AC mutant (squares) cells were grown at the indicated temperature and CO_2_ supply under 40 µmol photons m^−2^ s^−1^. The inserts show the same data using a different scale. Growth was monitored by measuring the optical density at 730 nm (OD_730_). The values represent the mean ± SD of three independent experiments.
(EPS)

Figure S3 **Changes in the Q_A_^−^→Q_B_ electron transfer rates upon incubation at 43°C.** AC mutant cells growing at 30°C for 3 days were transferred to 43°C and their Q_A_^−^→Q_B_ electron transfer rate was measured at the indicated times (See Materials and methods). The values represent the mean ± SD of three independent experiments.
(EPS)

Figure S4 **D1 and Rubisco proteins immunoblot signal response.** A dilution series of ΔKS (circles) and AC (squares) thylakoid membrane samples was loaded in SDS-PAGE and subsequently immunoblotted using specific antibodies raised against D1 (A) and Rubisco (B) proteins. Immunoblot signal changes were quantified by integrating variable pixel intensity from scanned blots. The values were normalized to the sample containing 1 µg of chlorophyll. The data represent the mean ± SD of three independent experiments.
(EPS)

Table S1 **Thylakoid membrane lipids composition in** ***Synechocystis*** **sp. PCC6803 and** ***Thermosynechococcus elongatus*** **grown at the indicated temperatures.** Lipids were extracted from thylakoid membranes. Lipid classes were separated by thin-layer chromatography, sprayed with a primuline solution and quantified by integrating variable pixel intensities of the scanned plates. The values represent the mean of three independent experiments. The deviation of values was within ± 2%. Monogalactosyldiacylglycerol, MGDG; digalactosyldiacylglycerol, DGDG; phosphatidylglycerol, PG; sulfoquinosyldiacylglycerol, SQDG.
(DOC)
