# Supplementary material for: Double Mutation in Photosystem II Reaction Centers and Elevated CO2 Grant Thermotolerance to Mesophilic Cyanobacterium
Source: PLoS One. 2011 Dec 22;6(12):e28389. doi: 10.1371/journal.pone.0028389 (PMC3245225; doi:10.1371/journal.pone.0028389)
Supplement: Table S1 — Thylakoid membrane lipids composition in Synechocystis sp. PCC6803 and Thermosynechococcus elongatus grown at the indicated temperatures. Lipids were extracted from thylakoid membranes. Lipid classes were separated by thin-layer chromatography, sprayed with a primuline solution and quantified by integrating variable pixel intensities of the scanned plates. The values represent the mean of three independent experiments. The deviation of values was within ± 2%. Monogalactosyldiacylglycerol, MGDG; digalactosyldiacylglycerol, DGDG; phosphatidylglycerol, PG; sulfoquinosyldiacylglycerol, SQDG. (DOC) [file pone.0028389.s005.doc]

| **Cyanobacterium** | ***Synechocystis* sp. PCC 6803** | | | | ***T. elongatus*** |
| --- | --- | --- | --- | --- | --- |
|
| **Growth temperature** | **30** °**C** | | **43** °**C** | | **57** °**C** |
| **Strain** | ΔKS | AC | ΔKS | AC | WT |
| **MGDG [%]** | 22.8 | 18.9 | 35.6 | 36.2 | 50.6 |
| **DGDG [%]** | 34.5 | 41.1 | 28.7 | 32.2 | 30.4 |
| **SQDG [%]** | 28.8 | 28.2 | 25.2 | 22.4 | 15.4 |
| **PG [%]** | 13.8 | 11.9 | 10.5 | 9.2 | 3.6 |

**Table S1**. Thylakoid membrane lipids composition in *Synechocystis* sp. PCC6803 and *Thermosynechococcus elongatus* grown at the indicated temperatures.

Lipids were extracted from thylakoid membranes. Lipid classes were separated by thin-layer chromatography, sprayed with a primuline solution and quantified by integrating variable pixel intensities of the scanned plates. The values represent the mean of three independent experiments. The deviation of values was within ± 2%. Monogalactosyldiacylglycerol, MGDG; digalactosyldiacylglycerol, DGDG; phosphatidylglycerol, PG; sulfoquinosyldiacylglycerol, SQDG.
